# Myxedema Coma: A Forgotten Medical Emergency With a Precipitous Onset

**DOI:** 10.7759/cureus.10478

**Published:** 2020-09-16

**Authors:** Roshan Acharya, Ce Cheng, Michael Bourgeois, John Masoud, Edwin McCray

**Affiliations:** 1 Internal Medicine, Cape Fear Valley Medical Center, Fayetteville, USA; 2 Internal Medicine, Campbell University School of Osteopathic Medicine, Fayetteville, USA; 3 Internal Medicine, Banner University Medical Center - South Campus, Tucson, USA; 4 Internal Medicine, Campbell University School of Osteopathic Medicine, Buies Creek, USA

**Keywords:** myxedema coma, severe hypothyroidism, malnutrition

## Abstract

Myxedema coma is a rare life-threatening disorder characterized by severe hypothyroidism leading to multiorgan failure and even death. This case also reminds clinicians that the misnomer “coma” is misleading, and the patient can present with less severe symptoms. We present a case of a 72-year-old female with a history of primary hypothyroidism who presented to the emergency department with progressively worsening confusion for three days. Laboratory results revealed thyroid-stimulating hormone (TSH) 402.0 µU/L and free thyroxine (T4) 0.22 ng/dL. The patient was compliant with the levothyroxine but she found to be malnourished on presentation. The patient was treated with intravenous levothyroxine and liothyronine. The patient’s mental status improved to the baseline, and she was discharged to a skilled nursing facility. Myxedema coma is a rare but life-threatening disorder that providers should be familiar with, including management and treatment. To the best of our knowledge, this is the highest TSH level ever reported so far, and the first case of myxedema coma precipitated due to malnutrition.

## Introduction

Myxedema coma (MC) is a rare disorder characterized by severe hypothyroidism, presenting as altered mental status, hypothermia, and a decrease in the function of multiple organ systems. MC is a life-threatening condition with a high mortality rate; rapid recognition is critical to avoid end-organ damage. A retrospective observational study reported the incidence of MC at 1.08 per million per year, with a mortality rate of 29.9% [1]. The mortality rate can be high as 60% even with the best possible treatment [2]. Fortunately, due to the wide availability of assays to measure thyroid-stimulating hormone (TSH) and medications necessary to treat hypothyroidism, the presentation of MC is now rare.

The symptoms of MC are non-specific and include hypotension, hypothermia, hyponatremia, hypoglycemia, bradycardia, and myxedema (soft tissue edema secondary to the deposition of mucopolysaccharides) of the hands and face. Neurologic symptoms include lethargy and obtundation. Despite the name, coma is an uncommon presentation and is not necessary to make the diagnosis [2]; its presence is a poor prognostic indicator [3].

## Case presentation

A 72-year-old female with a medical history of primary hypothyroidism presented to the emergency department for progressively worsening confusion for three days. Her husband reported that her oral intake had been getting slowly poor over a period of few months and for past few weeks she has been eating very less. He had also noticed difficulty maintaining balance while ambulating for the past few weeks. He confirmed he had been giving her thyroid medicine. Initial workup in the emergency department revealed hypothermia with a rectal temperature of 93.7°F, an irregularly irregular heart rate at 55 beats per minute, blood pressure of 160/108 mmHg, respiratory rate was 8 breaths per minute, oxygen saturation of 100% on ambient air, and a body mass index (BMI) of 19. On physical exam, the patient was ill appearing with marked macroglossia. Bowel sounds and breath sounds were decreased. The neurologic exam revealed diffuse hypertonicity of all four extremities. Deep tendon reflexes were reduced to 1+ globally. Glasgow Coma Score was 12/15. The complete blood count was unremarkable. The comprehensive metabolic panel was significant for hyperkalemia with a potassium of 5.7 mmol/L, albumin level of 1.8 g/dL, as well as acute kidney injury with blood urea nitrogen (BUN) 34 mg/dL and creatinine 1.86 mg/dL. The blood glucose level was 89 mg/dL. The arterial blood gas revealed a pH of 7.36, pO_2_ 64.5, and pCO_2_ of 38.8. Troponin was <0.015 ng/mL. Electrocardiography (EKG) revealed new-onset atrial fibrillation at a rate of 65 per minute without peaked T waves. A plain CT scan of the head and CT angiogram of the head and neck revealed no acute intracranial bleeding, ischemia, or significant stenosis in major neck vessels. Urinalysis was not suggestive of infection and a urine drug screen was negative. TSH was significantly elevated at 402.0 µU/L and the free thyroxine (T4) was low at 0.22 ng/dL. Cortisol level was 16.1 µg/dL but it was not a morning cortisol level since the patient presented in the evening. Further inquired history later on revealed that the patient’s primary hypothyroidism had been very difficult to control even with compliance and follow up for the past few months. The most recent TSH two months prior to the admission was 180 µU/L, so her levothyroxine regimen was increased to 112 mcg/daily from 100 mcg/daily by her primary care physician. 

A diagnosis of MC was made. The patient was admitted to the step-down unit for the management of MC. She was given intravenous loading doses of intravenous hydrocortisone 100 mg, levothyroxine 100 mcg, and liothyronine 10 mcg by the admission team, and then started on intravenous levothyroxine 100 mcg daily, liothyronine 2.5 mcg every eight hours, and hydrocortisone 100 mg every eight hours. Hyperkalemia was resolved with IV insulin and glucose and oral polystyrene sulfonate. Calcium gluconate was also given in the emergency department for cardioprotection. 

Her clinical condition improved over the next two days, and the patient gradually regained the ability to speak and interact with hospital staff appropriately. Given elevated morning cortisol level 76.9 µg/dL and neurological improvement, IV hydrocortisone was reduced to 50 mg every eight hours. However, she continued to be hypothermic with a temperature of 94.7°F. Blood cultures obtained on the admission day returned positive for gram-positive cocci in clusters in both bottles. Concerning for sepsis, she was started on intravenous vancomycin for suspected bacteremia; a warming blanket was applied to raise her body temperature. On hospital day 3, she suddenly became irritable, uncooperative, and continually attempted to get up from bed throughout the night despite repeated requests by the nursing staff to remain in bed. She was unsteady, and she pulled out all her peripheral intravenous lines. She ultimately had to be restrained with a vest and soft mitts. This was also thought to be the continuum of MC. On hospital day 4, the patient’s cognition improved significantly. Laboratory results revealed improved free triiodothyronine (T3), free T4, and morning cortisol level. The patient was deescalated from the step-down unit to a regular medicine floor. The thyroid replacement regimen transitioned to oral levothyroxine 200 mcg daily, while IV cortisone was continued. On hospital day 5, the previous blood cultures ultimately identified the organisms mixed with alpha-hemolytic Streptococcus and coagulase-negative Staphylococcus, consistent with culture contamination. Also, repeat blood cultures revealed negative results. Therefore, vancomycin was discontinued. 

The patient continued to exhibit poor appetite and low blood sugar despite treatment with dronabinol for appetite stimulation and scheduled oral glucose tablets for hypoglycemia. Adrenal insufficiency was ruled out given a repeat normalized morning cortisol, for which IV hydrocortisone was discontinued on hospital day 11. Due to the patient’s continuing refusal to eat, a percutaneous endoscopic gastrostomy (PEG) tube was placed. She tolerated regular tube feeds and her hypoglycemia resolved. Ultimately, the patient was discharged in stable condition with the improvement of her mental status to the baseline to a skilled nursing facility, with a regimen of oral levothyroxine 112 mcg daily. At one-month follow-up upon discharge, she continued to improve clinically. She was self-ambulatory and tolerated oral feeds well. The plan for removing the PEG tube had been made. Her TSH was reported to be 12.5 µU/L. 

## Discussion

Primary MC is unusual, though in rare cases patients will present without an underlying diagnosis of hypothyroidism. About 95% of all MC cases are due to primary hypothyroidism [4]. The diagnosis of MC can be difficult due to a delay in seeking medical treatment and the non-specific symptoms of hypothyroidism, and many patients do not actually present in a coma [2]. Patients may present MC with less severe symptoms of hypothyroidism, which requires prompt consideration and treatment of this condition to avoid cardiopulmonary deterioration, given its high morbidity and mortality. A diagnostic scoring system has been developed for MC, and our patient scored 80 which was highly suggestive of MC [5]. 

Rapid recognition and treatment are critical to prevent permanent end-organ damage and death, as early treatments and interventions of MC can reduce the mortality rates to about 20%-25% from quoted mortality rates of 60%-70% [2]. Therefore, identifying any precipitating factors is critical to the management of MC. Common examples of such factors include infection, prolonged cold exposure, surgery, and sedating drugs. Infection and septicemia are usually the leading precipitating factors. MC is more common in elderly females with long-standing undiagnosed hypothyroidism [4], and the most common reason of failure oral thyroid therapy is non-compliance [6]. Since levothyroxine has a long half-life, missing one dose of levothyroxine can influence on the TSH and thyroid hormone levels for several days [7]. 

MC appears more common among elderly women with long-standing pre-existing hypothyroidism [4], who often have gastrointestinal disorders that might impair thyroxine absorption, even though they have been treated with thyroxine replacement therapy [8]. In our case, infection, septicemia, non-compliance, and other causes had been ruled out, and we believe that poor nutritional status as evidenced by very low serum albumin and low BMI was the primary reason for severe hypothyroidism. This was also supported by the rapid improvement in her condition with the help of intravenous levothyroxine and continued improvement while getting nutritional support through the PEG tube. Although she was compliant with medicine, her long-standing malnourished state had caused a chronic refractory hypothyroid state which possibly precipitated MC eventually. Virili et al. discussed that “gastrointestinal malabsorption of oral T4 represents an emerging cause of refractory hypothyroidism and may be more frequent than previously reputed.” Gastric and intestinal disorders including Helicobacter pylori infection, celiac disease, and lactulose intolerance, prior bariatric surgery, nutrition, food, and drug interference with levothyroxine could lead to refractory hypothyroidism [8]. Thus, a further workup for gastrointestinal malabsorption might be warranted for some patients who are compliant with medications but still do not achieve well-controlled hypothyroidism. 

Due to the rarity of MC, there is a lack of evidence-based treatment regimens supported by adequate randomized clinical trials [9,10]. Generally, management of MC includes thyroid hormone replacement, glucocorticoids, treating the underlying cause, and supportive measures such as fluid resuscitation and warming [10]. The current recommendations published by the American Thyroid Association (ATA) recommend a loading dose of glucocorticoids followed by T4 replacement, with T3 replacement as an optional adjunct in select patients. Treatment begins with empiric coverage for adrenal insufficiency with glucocorticoids. Options are intravenous dexamethasone 2-4 mg every 12 hours or intravenous hydrocortisone 50-100 mg every 8 hours. Notably, dexamethasone does not interfere with an adrenocorticotropic hormone (ACTH) stimulation test and is thus often preferred over hydrocortisone [10]. A loading dose of intravenous levothyroxine at 200-400 mcg is then given, followed by a daily dose of intravenous levothyroxine defines at 75% of 1.6 mcg/kg/day (the lower dose is given due to the parenteral route to avoid overtreatment). 

T3 replacement with intravenous liothyronine is considered an optional adjunct since the T4 conversion to T3 may be impaired, especially in elderly patients with MC. Also, it is vital to quickly reverse intracellular T3 deficiency as it can lead to cardiogenic shock, hypoxia, and coma [2,10]. This is the reason why we gave T3 in this case. But T3 replacement should be avoided in patients with cardiac dysfunction, i.e. heart failure. If pursued, T3 replacement starts with a loading dose of 5-20 mcg, followed by 2.5-10 mcg every eight hours [10]. Parenteral administration is favored in the early stage of treatment to increase the effectiveness of the treatment and bypass a gastrointestinal tract that is less functional in the setting of severe hypothyroidism. 

As the patient’s clinical condition improves, levothyroxine can be switched to the oral route at the full 1.6 mcg/kg/day dose; liothyronine, if given, should be discontinued at this point [10]. Thyroid hormones may be measured every 24 to 48 hours monitoring responses to thyroid hormone replacements. Unfortunately, due to the rarity of the disease, there is a dearth of evidence available; the ATA guidelines are therefore based largely on expert opinion and are not intended to replace clinical judgments or individual decision-making. Due to the unpredictability of disease and lack of a definitive treatment regimen, following the patient’s clinical progression and adjusting the management accordingly appears to be the most advantageous strategy.

The risks of aggressive thyroid hormone treatment are significant since rapid administration may precipitate myocardial infarction or fatal arrhythmias [11]. This is particularly relevant in this case, as this patient initially presented in atrial fibrillation [12]. Based on the ATA guidelines, we chose a combination therapy of hydrocortisone, levothyroxine, and liothyronine. We chose to pursue adjunct therapy with liothyronine because T3 is more potent than T4 and the peripheral conversion of T4 to T3 may be impaired in MC patients. Notably, T3 treatment alone may be associated with high mortality [9]. Additionally, some studies have suggested a higher mortality rate associated with levothyroxine doses higher than 500 mcg daily and liothyronine doses higher than 75 mcg daily [13].

## Conclusions

Although MC is rare nowadays, it is a life-threatening medical emergency with a high mortality rate even with appropriate and timely treatments. High suspicion is needed as symptoms are usually vague and non-specific and it can mimic other conditions, such as septic shock. It is important to keep in mind that malnutrition may lead to severe hypothyroidism. Providers must be careful while proving care to an elderly, hypothyroid patient who is at risk for malnutrition as it might lead to MC. Early detection can lead to early treatment and avoid unnecessary workup. 
